# Tumors of the Digestive System: Comprehensive Review of Ancillary Testing and Biomarkers in the Era of Precision Medicine

**DOI:** 10.3390/curroncol30020182

**Published:** 2023-02-16

**Authors:** Attila Molnar, Hunter Monroe, Hasan Basri Aydin, Mustafa Erdem Arslan, Andrea Lightle, Hwajeong Lee, Tony El Jabbour

**Affiliations:** 1 Department of Pathology, Icahn School of Medicine at Mount Sinai, New York, NY 10025, USA; 2 Department of Pathology, West Virginia University, Morgantown, WV 26506, USA; 3 Department of Pathology, Albany Medical Center, Albany, NY 12208, USA; 4 Department of Pathology, Memorial Sloan Kettering Cancer Center, New York, NY 10065, USA

**Keywords:** digestive system cancer, esophageal tumors, gastric tumors, pancreatic tumors, hepatobiliary tumors, colorectal tumors, immunotherapy, biomarkers, ancillary tests

## Abstract

Immunotherapy has remained at the vanguard of promising cancer therapeutic regimens due to its exceptionally high specificity for tumor cells and potential for significantly improved treatment-associated quality of life compared to other therapeutic approaches such as surgery and chemoradiation. This is especially true in the digestive system, where high rates of mutation give rise to a host of targetable tumor-specific antigens. Many patients, however, do not exhibit measurable improvements under immunotherapy due to intrinsic or acquired resistance, making predictive biomarkers necessary to determine which patients will benefit from this line of treatment. Many of these biomarkers are assessed empirically by pathologists according to nuanced scoring criteria and algorithms. This review serves to inform clinicians and pathologists of extant and promising upcoming biomarkers predictive of immunotherapeutic efficacy among digestive system malignancies and the ancillary testing required for interpretation by pathologists according to tumor site of origin.

## 1. Introduction

Digestive system (DS) cancers constitute a significant portion of the global cancer burden, accounting for up to 26% of aggregate cancer incidence according to recent estimates while serving as the leading cause of cancer mortality in aggregate [1,2,3]. Although these statistics are no doubt dire and concerning, the understanding of molecular pathways critical to the development of these cancers has opened the door to the new era of precision medicine and immunotherapy. Over the last few decades, attacking a target at the molecular level via immune-related strategies has become the mainstay of cancer therapeutics [4]. Moreover, due to their mitigated side-effect profiles, these therapies are increasingly incorporated in the routine clinical practice. However, this advent came with expansive additions to the workflows of cancer diagnosis and treatment. Consequently, the contemporary “precision medicine” approach entails finding the possible therapeutic targets (also called biomarkers) via additional laboratory tests (also called ancillary tests) to guide immunotherapy. Biomarkers predictive of successful response to immunotherapy have become an integral part of this “precision medicine” approach. Being at the intersection of diagnosis and treatment, and having access to preserved tumoral tissue, pathologists are now asked to perform, interpret, and report the vast majority of ancillary tests available. Herein, we review the scientific concepts behind current immunotherapeutic strategies and ancillary tests. We also expand on the interpretation and pitfalls of commonly used tests. More importantly, we provide an organ-based guide to biomarkers, their companion diagnostic tests, and their relevance to clinical practice for major digestive tumors arising from the esophagus, stomach, intestines, pancreas, hepatobiliary system, and anus, as well as other miscellaneous tumors not relegated to a specific site of origin.

## 2. Immunotherapeutic Agents Applicable to Digestive System Tumors

### 2.1. Immune Checkpoint Inhibitors (ICIs)

Because the immune response serves as the most effective endogenous first-line response to nascent cancers, tumor cells have taken to shifting the balance of immune signals from pro- to-anti-inflammatory, thereby favoring an immunosuppressive tumor microenvironment (TME) that is more hospitable to the developing tumor. To accomplish this, tumor cells may rely on anti-inflammatory checkpoint proteins including the programmed cell death receptor-1 (PD-1) and programmed cell death ligand-1 (PD-L1) complex found on T-cells and tumor cells, as well as the cytotoxic T lymphocyte-associated molecule-4 (CTLA-4) present on the surface of activated T-cells that serve to regulate T-cell tolerance under normal circumstances [5]. These checkpoint proteins exert their anti-inflammatory effects by preventing the binding of T-cell costimulatory receptors to their endogenous ligands (e.g., CTLA-4 disrupts the interaction between CD28 on T-cells and the B7 protein found on antigen-presenting cells). Overexpression of checkpoint proteins by tumor cells or T-cells in the TME will result in recruitment of immunosuppressive regulatory T-cells, downregulation of tumor antigen expression, and induction of T-cell tolerance or apoptosis, thus negatively regulating T-cell-mediated activity and facilitating tumor proliferation and metastasis [5].

Inhibition of these checkpoint proteins by monoclonal antibodies referred to as “immune checkpoint inhibitors (ICIs)” serves as an effective therapeutic approach by decreasing the threshold for T-cell activation and reinvigorating the antitumoral immune response [5]. Although a bevy of ICIs are currently employed or under investigation for use in solid and hematopoietic tumors from many sites, the PD-1 inhibitor, pembrolizumab, is perhaps the most well-known, as it has proven successful for the management of unresectable or metastatic mismatch repair-deficient (dMMR) or high-microsatellite-instability (MSI-H) colorectal carcinoma [6]. Only a minority of patients (20–40%) show a detectable response to ICIs due to the expression of critical biomarkers such as PD-L1 on T-cells [7], however, necessitating further screening with biomarkers to assess for eligible patients.

### 2.2. Adoptive T-Cell Transfer (ACT)

Adoptive cell therapy (ACT) refers to the intravenous transfer of immune cells, classically tumor-resident or modified peripheral blood T-cells, into cancer patients to stimulate a potent antitumoral immune response. T-cell transfer may be classified into three types: resident tumor-infiltrating lymphocytes (TILs), T-cells with modified T-cell receptors (TCRs), and T-cells with chimeric antigen receptors (CARs) [8]. In the first case, TILs are extracted from resected tissue and introduced to patients following endogenous lymphodepletion, an approach that is widely used for the management of melanomas [8]. In the second modality, peripheral blood T-cells are transduced to express modified TCRs with high specificity for tumor antigens. This approach, however, remains dependent on expression of major histocompatibility complex (MHC) by tumor cells. To enable MHC-independent target recognition, CAR-modified T-cells harbor monoclonal antibodies that serve as the antigen-binding domain, thus bypassing the need for MHC [8]. The final approach has proven successful in the treatment of hematopoietic neoplasms, and trials against solid tumor antigens such as CEA, mesothelin, and GUCY2C in colorectal carcinoma are promising [9,10].

Although not yet instituted in regular clinical practice, CAR natural killer (NK) cells may provide the added benefits of short-lived responses, lack of an HLA matching prerequisite, and reduced risk of side-effects such as cytokine release syndrome [11]. Incubation of peripheral blood lymphocytes with select cytokines such as interferon-γ (IFN-γ) and interleukin-2 (IL-2), as well as anti-CD3 antibody induces a CD3^+^CD56^+^ subset of lymphocytes with mixed T-cell and NK cell activity (cytokine-induced killer or CIK cells) that possess potent antitumor cytotoxic activity for tumor cells expressing and lacking MHC [12]. Although still in early stages of development, CIK adoptive cell therapy has demonstrated promising results in many digestive system malignancies such as gastric adenocarcinoma and hepatocellular carcinoma [12].

### 2.3. Vaccine Based Immunotherapy

Although traditionally used as a prophylactic measure for infectious etiologies, vaccine efficacy as an immunotherapeutic modality in the management of various cancers is a burgeoning subject of research. These cancer-targeting vaccines operate in a similar manner to prophylactic vaccines in that their goal is to incite an effective humoral (antibody-based) or cell-mediated (T-cell-reliant) immune response that eliminates tumor cells with minimal damage to non-neoplastic host cells [13]. Cancer vaccines are reliant on the targeting of suitable tumor-associated antigens (TAAs), which should ideally be (a) expressed on the surface of all or the majority of tumor cells, (b) minimally expressed by non-neoplastic cells, (c) necessary for tumor cell survival, and (d) capable of eliciting a satisfactorily robust immune response [13,14,15]. Current vaccination-based immunotherapy relies on three overarching vaccine subtypes: cellular vaccines (inoculation of whole autologous or allogeneic tumor cells or of TAA-loaded dendritic cells (DCs)); protein or peptide subunit-based vaccines with specificity for selected TAAs; genetic (nucleic acid-based or virus-loaded) vaccines. All of these are defined by their respective vectors for cell entry and are chosen due to their unique advantages and disadvantages such as cost and timeliness of production, spectrum of targeted TAAs afforded, and immunogenicity [14]. A novel DC vaccine has been shown to inhibit pancreatic cancer metastases via intraperitoneal injections [16].

### 2.4. Indolamine 2,3 Dioxygenase Inhibitors (IDOs)

Indolamine 2,3-dioxygenase (IDO) is an enzyme that catalyzes the rate-limiting step in the conversion of 95% of tryptophan to kynurenine, contributing to an immunosuppressive TME by upregulating PD-1 expression in CD8^+^ TILs, stimulation of Tregs and myeloid-derived suppressor cells (MDSCs), and perhaps even inducing neovascularization [17,18]. IDO inhibitors (IDOIs) are currently under investigation as a secondary therapeutic option when ICI monotherapy proves ineffective. High IDO expression in the TME of colorectal cancer (CRC) is associated with poor outcomes even in the presence of high CD8^+^ TIL counts [19]. The IDO1-competitive antagonist epacadostat, initially developed for the treatment of advanced-stage melanoma, is currently being investigated in tandem with chemoradiation in an ongoing clinical trial for the management of locally advanced rectal cancer [17].

### 2.5. CCL2–CCR2 Signaling Pathway Inhibitors

Chemokines of the CC family, particularly interactions between the CCL2 ligand and its receptor CCR2, are believed to contribute to an immunosuppressive TME. CCL2–CCR2 immunosuppressive sequelae include the transition of tumor-associated macrophages (TAMs) to M2-type macrophages, recruitment of regulatory T-cells with enhanced production of suppressive cytokines such as IL-10, inhibition of apoptosis and autophagy in the setting of nutrient deprivation, and facilitation of tumor migration via upregulation of matrix metalloproteinase-9 (MMP-9) [20]. Moreover, studies have demonstrated that up to 100% of solid cancers express CCR2, and a further large proportion of tumors produce CCL2 [20], including hepatocellular carcinoma [21], pancreatic carcinoma [22], gastric adenocarcinoma [23], and colorectal carcinoma [24]. Inhibitors of this chemokine pathway have shown promising results, especially when used concomitantly with radiotherapy or chemotherapy, both of which may increase the number of CCR2^+^ immunosuppressive macrophages in the TME [25,26]. Of note, one clinical trial observed objective tissue response and local control in 97% of patients with advanced-stage or borderline resectable pancreatic cancer when the PF-04136309 CCR2 antagonist was paired with the FOLFIRINOX chemotherapeutic regimen, a result not found with FOLFIRINOX monotherapy [25].

## 3. Ancillary Tests Applicable to Digestive System Tumors

### 3.1. Immunohistochemistry (IHC)

Immunohistochemistry (IHC) refers to the use of antibodies to determine tissue distribution of a complementary antigen of interest as assessed on microscopic examination by a pathologist. This technique relies on tissue sections obtained from biopsies that are formalin-fixed paraffin-embedded (FFPE) or subject to a similar mode of fresh tissue processing [27]. Various enzymes or other chemicals are used to label antigen–antibody binding by producing either chromatic reactions (chromogenic IHC) or fluorescent dyes (immunofluorescence) [27]. IHC is ubiquitously used in surgical pathology to establish a diagnosis where standard histomorphologic examination of hematoxylin-and-eosin (H&E)-stained slides is insufficient; it is also used to identify prognostic markers indicative of the tumor’s biology and to determine the anticipated response to treatments via quantitative detection of predictive biomarkers. Examples of this include the CD8^+^ TIL density/ImmunoScore and evaluation of PD-1/PD-L1 expression [28].

### 3.2. Fluorescence In Situ Hybridization (FISH)

Fluorescence in situ hybridization (FISH) allows for direct analysis of genetic material at chromosomal and single-gene levels. FISH is accomplished by hybridizing selected DNA strands impregnated with fluorophore-labeled nucleotides (probes) onto complementary DNA sequences of interest in cells or tissue for visualization under a fluorescence microscope or other high-throughput imaging system [29]. The high analytical resolution (100–200 kilobase pairs (kb) versus 5–10 megabase (Mb) pairs of the now antiquated karyotyping technique) affords the identification of recurrent microinsertions, microdeletions, and rearrangements/translocations in solid tumor cell tumors at any phase of the cell cycle, thus serving as an invaluable tool in the diagnosis, prognostic evaluation, and assessment of post-therapeutic responses in patient tumor cells [29]. Of note, a newer rendition of FISH allows for the detection of heterogeneous mRNA expression between tumor cells and non-neoplastic cells, which has promoted the development of reliable biomarkers such as albumin mRNA for neoplasms of hepatic origin [30].

### 3.3. Polymerase Chain Reaction (PCR)

Polymerase chain reaction (PCR) is a technique used to amplify targeted DNA sequences. PCR requires a DNA template (the gene to be amplified), a heat-resistant thermus aquaticus (Taq) polymerase, DNA primers, complementary deoxynucleotide triphosphates (dNTPs), and a chemical buffer containing cationic cofactors; it consists of repeated (20–40) thermal cycles of denaturation, annealing, and elongation of DNA strands [31]. One modality, quantitative (also called real-time) PCR (qPCR/RT-PCR), can be performed to identify mutations and copy number variations within genes of clinical relevance [30]. This has been used extensively to assess for the presence of mutations such as KRAS, EGFR, or ALK that may be subject to targeted therapy in certain cancers [32]. Another modality, digital PCR (dPCR), uses multiple dilutions of the parent genetic sample to provide more precise measurements of nucleic acid content, which may be used to distinguish whether clinically actionable mutations are localized to a single allele or present in both alleles of a given gene. This is useful for the determination of copy number variants (CNVs) and point mutations [31].

### 3.4. Next-Generation Sequencing (NGS)

Next-generation sequencing (NGS) provides parallel sequencing of massive swathes of DNA using bioinformatic analyses to map fragments according to the human reference genome, permitting the study of entire cancer genomes to facilitate the diagnosis and prognostic evaluation of cancers, as well as the identification of targetable causal mutations [32,33]. Many variations of this technique are used in clinical practice or are currently undergoing extensive clinical research. Whole-exome sequencing (WES) identifies only mutations found in the coding regions (exons) of genes that directly contribute to protein synthesis and function [34]. Whole-genome sequencing (WGS), on the other hand, includes exons, as well as mutations within noncoding regions (introns) that affect downstream gene expression and splicing [34]. Germline testing with concomitant sequencing is ordered when a heritable mutation is suspected and identifies pathogenic germline variants (PGVs), which may be present in >30% of mutations detected by sequencing according to a recent study [35].

Of special importance to clinical practice is targeted genomic sequencing (TGS), which elucidates the presence of a panel of pathogenic genes or targets known to initiate clinically relevant disease (driver mutations) or that are clinically actionable, thereby helping to stratify patients into risk groups on the basis of mutational status. Because of the high sequencing depth of this approach, TGS may be used on FFPE tissue or circulating tumor DNA (ctDNA) where DNA preservation or tumor content is poor [36]. Of note, ctDNA is among the most prominent liquid biopsy-based technologies utilized for TGS due to its ability to noninvasively measure tumor genetic profiles and, thus, facilitate tumor diagnosis, prognosis, and evaluation of postoperative/post-treatment recurrence when tumors have metastasized and rendered tissue sample obtainment by surgery or conventional biopsy arduous or impossible [33]. TGS allows for the discovery of subclonal mutations present in a minority of malignant cells [36] and identification of mutations associated with resistance to certain targeted therapies such as HER2 in breast and esophagogastric cancers or KRAS mutations conferring resistance to anti-EGFR therapy in colorectal cancer [33].

## 4. Interpretation and Reporting of Ancillary Tests for Relevant Biomarkers of Digestive System Tumors

### 4.1. HER2

Human epidermal growth factor receptor (HER2) is a tyrosine kinase receptor encoded by the ERBB2 gene that is strongly implicated in tumor proliferation, differentiation, growth, and abrogation of apoptosis when amplified or overexpressed [37,38,39]. HER2 is frequently overexpressed in gastroesophageal cancers (GECs); thus, HER2 testing by IHC or ISH is recommended for all patients with advanced, unresectable GECs [37]. The anti-HER2 monoclonal antibody, trastuzumab, may be employed in this subset of patients and confer significant survival benefit [37,39]. Moreover, immunotherapies such as ICIs have been FDA-approved for all patients with HER2^+^ GECs with concomitant chemotherapy [40], and the KEYNOTE-859 trial is currently investigating the use of immunotherapy in GECs with negative HER2 status [41].

HER2 assessment by IHC relies on the four-tiered Hoffman scoring system consisting of negative (scores 0 to 1+), equivocal (2+), and positive (3+). The scoring criterion varies depending on whether the tumor in question is of the gastroesophageal adenocarcinoma (GEA) or squamous cell carcinoma (SCC) subtype, and whether surgical resection specimens or biopsies are provided for testing. In the latter scenario, it is recommended that at least five biopsy specimens be collected for accurate HER2 assessment [37]. In contrast to breast cancers which require complete membranous staining of tumor cells, HER2 staining in GEAs is interpreted as positive if incomplete membranous staining of any manner, such as lateral or basolateral, is identified (see Table 1) [37,40]. SCCs, on the other hand, demonstrate a nearly identical staining pattern to breast cancers and should, therefore, be scored using the traditional system developed for breast cancers (see Table 2) [38].

Those samples scored as equivocal (2+) should be followed with ISH techniques such as FISH, brightfield ISH, or dual-color in situ hybridization (DISH) before clinical action is undertaken per NCCN guidelines [37,38]. ISH testing of GEC specimens requires 20 nonoverlapping tumor cell nuclei for enumeration of HER2 and/or CEP17 probes [37]. Nuclei should be counted within areas of strongest HER2 staining by IHC marked by the pathologist. A ratio of HER2/CEP17 probes of 2.0 or more is read as positive for HER2, while a ratio less than 2.0 is considered negative [37]. ISH may additionally be interpreted as positive if the ratio of HER2/CEP17 probes is less than 2.0, but if >6 HER2 and >3 CEP17 signals are visualized [37]. If ISH and IHC are uninterpretable, genomic testing strategies such as droplet digital PCR may be instituted [37]. For a summary of HER ISH interpretation, see Table 3.

### 4.2. PD-L1

**Figure 1:**
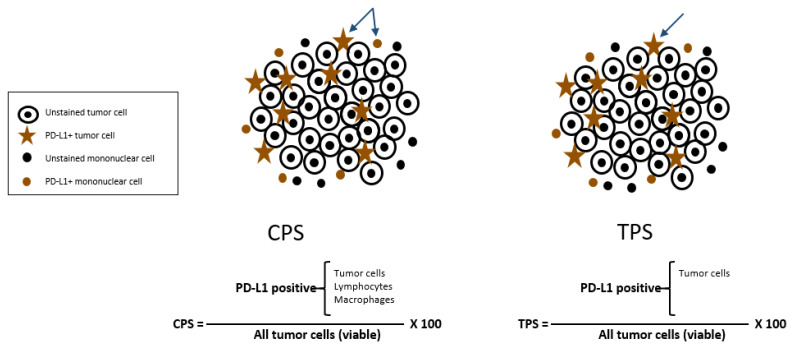
Schematic representation of the available scoring criteria for PD-L1 assessment.

As previously discussed, PD-L1 is a major checkpoint protein expressed on the surface of tumor cells. Because of its significance to cancer survival and the utility of ICIs that is contingent upon its presence, direct testing for PD-L1 is frequently performed via IHC on tumor specimens. PD-L1 may be calculated as the tumor proportion score (TPS), which enumerates PD-L1 expression exclusively on tumor cells, or the combined proportion score (CPS), which includes tumor cells, lymphocytes, and macrophages (see Figure 1) [42,43]. Because GI cancers predominantly contain more immune cells expressing PD-L1, it was previously recommended that pathologists only document the CPS in their pathology reports. As nivolumab is now indicated for ESCC with TPS ≥ 1, TPS should also be reported for esophageal squamous cell cancers (ESCCs).

The cutoff for identifying meaningful responses to ICIs is subject to variability depending on the PD-L1 antibody clone used for IHC and the site of interpretation. Many clones may be used to assess PD-L1; however, the FDA has currently approved three clones: Dako 28-8, Ventana SP142, and Dako 22C3, the latter of which may be used to assess solid tumors in many sites including the GI tract [42]. Because the concordance of results among these clones is inconsistent [42], the clone used should be documented in the pathology report.

At present, PD-L1 cutoffs for GI tumors are only established in the upper GI tract and are stratified according to the choice of treatment with pembrolizumab or nivolumab. For adenocarcinomas of the esophagus or gastroesophageal junction (GEJ), CPSs ≥5 and ≥10 indicate the option of treatment with nivolumab and pembrolizumab, respectively. In ESCCs, pembrolizumab is indicated for patients with CPS ≥ 10 and nivolumab for TPS ≥ 1 (owing to the high proportion of PD-L1-expressing immune cells in ESCC). For gastric adenocarcinomas, nivolumab is indicated for patients with CPS ≥ 5 [42]. These clinical recommendations are summarized in Table 4.

### 4.3. Microsatellite Instability (MSI)

Microsatellites refer to repetitive strands of DNA measuring 1–10 nucleotides that are highly unstable and prone to the accumulation of frequent (often thousands) mutations (microsatellite instability/MSI), both novel and germline [44,45]. These mutations are typically a result of impaired DNA repair mechanisms, especially mismatch repair (MMR), which comprises a complex system of proteins, of which the best characterized are MLH1, PSM2, MSH2, and MSH6. Defective MMR (dMMR) is a common source of MSI in DS malignancies, especially those of the colorectum, small bowel, and gastroesophageal region [46], where it may not only indicate the HNPCC syndrome (better known as Lynch syndrome) in the setting of heritable mutations, but also operate as a novel biomarker predictive of ICI efficacy, largely owing to the production of mutation-associated tumor neoantigens that may be recognized with high specificity and immunogenicity by tumor-infiltrating T-cells [44].

MSI/dMMR may be detected via IHC, PCR, or NGS in current clinical practice, all of which exhibit similar sensitivity and specificity but require nuanced incorporation into the diagnostic workup [45]. IHC testing of MLH1, PMS2, MSH2, and MSH6 is recommended for all DS cancers of the Lynch syndrome spectrum including adenocarcinomas of the gastroesophageal junction, small bowel, and colorectum. Mutations of these genes result in truncation or loss of that protein. As these proteins form heterodimeric relationships consisting of obligatory partners (MLH1 and MSH2) and secondary partners (PMS2 and MSH6), MLH1 and MSH2 mutations appear as lost nuclear staining of both the obligatory protein and its secondary counterpart; conversely, PMS2 and MSH6 mutations manifest as loss of the secondary proteins only [44,45,46,47].

Interpretation of MMR by IHC is nuanced, however, and necessitates careful review by pathologists. Isolated loss of MLH1 staining is uniquely associated with sporadic dMMR and should receive follow-up testing for the BRAF V600E mutation or MLH1 promoter hypermethylation associated with the CpG island methylator phenotype (CIMP) [48]. Methylation of this gene is among the most common causes of sporadic MSI and should be evaluated in the event of isolated MLH1 loss per IHC [48,49]. Although many laboratories may solely rely on BRAF mutation assays for the identification of sporadic MSI due to the strong proportional relationship between this marker and MLH1 methylation, follow-up may still be recommended given that up to one-third of patients with hypermethylated MLH1 lack BRAF mutations [49]. Currently available methods for MLH1 methylation include real-time PCR, methylation-specific PCR, pyrosequencing, and novel methylation arrays, which confer the additional benefit of detecting CpG sites in >95% of known genes simultaneously using a single batch of tests [48]. Loss of PMS2, MSH2, and MSH6 staining should be followed by genetic testing for Lynch syndrome. Retained nuclear staining of all MMR markers indicates a tumor classified as MSI-L or MSS.

IHC, however, is subject to several pitfalls that often require concomitant genetic testing for MSI. Preanalytical factors such as type of fixative or amount of time in fixative of processed tissue may affect the quality and pattern of MMR staining [50]. Aberrant staining patterns may render interpretation arduous or inconclusive and include focal staining due to hypoxia-induced proteolysis [47,49], lack of background positive internal control for comparison of MMR status [47,49], cytoplasm-restricted staining [47,49], defective MMR protein with positive staining due to missense mutations that retain reactive antigenicity [49], and absent MSH6 staining following neoadjuvant chemotherapy [45,49]. False-positive MMR staining may also be observed if obligatory partners such as MLH1 bind another secondary protein such as MSH3 [47]. See Table 5 for a summary of pitfalls associated with IHC.

Because of potential misinterpretation, genomic assays including PCR and less commonly NGS are recommended if IHC results are indeterminate or to exclude Lynch syndrome following negative PMS2, MSH2, and MSH6 staining and negative testing for BRAF V600E mutation. MSI-PCR compares the microsatellites between tumoral tissue and benign reference tissue to assess for MSI with high specificity. Currently, two major commercial panels, the Bethesda panel and quasimonomorphic panel [45], are used in clinical practice. Loss of at least two markers in either panel is interpreted as MSI-H, loss of one marker is interpreted as MSI-L, and loss of zero markers is read as MSS. NGS has recently entered clinical practice as an alternative to IHC or PCR, where it may sequence many microsatellites in addition to non-immunotherapeutic, targetable mutations such as KRAS [51]. NGS offers the advantage of eliminating the requirement of normal reference tissue and is recommended for MSI testing of GI cancers with sparse data regarding IHC and PCR efficacy such as pancreatic cancer or cholangiocarcinomas [45,46].

### 4.4. Tumor Mutational Burden (TMB)

Tumor mutational burden (TMB) uses NGS to quantify somatic mutations per million base pairs (Mb) of a given genetic sequence. This has practical implications in that high tumor cell mutagenicity is associated with the synthesis of tumor-specific neoantigens that may be targeted by tumor-infiltrating T-cells following immune induction by immunotherapies such as ICIs [52]. Due to the success of the KEYNOTE-158 clinical trial and studies showing improved median progression-free survival (PFS) and overall survival (OS) in patients with TMB-high tumors [53], in 2020, the FDA approved pembrolizumab monotherapy for all TMB-high solid tumors (defined as at least 10 mutations/Mb) that are refractory to other modes of treatment [52]. Thus, TMB has been established as a predictive biomarker of ICI response in a plethora of cancers, but especially those of the DS where mean TMB is routinely high (especially in colorectal and small intestinal adenocarcinomas), owing to the high prevalence of DNA repair mutations, and often strongly correlates with MSI-H status [52,54].

TMB calculation is influenced by several factors including tumor cell content, formalin fixation (which induces disruptive DNA crosslinks), cutoffs for high or low status, size of targeted panels and genes included therein, and choice of bioinformatics approach [52]. The gold standard of TMB assessment is WES, although TGS is largely preferred in clinical practice due to its lower cost, shorter turnaround times, high sequencing coverage, and inclusion of introns needed for gene fusion detection [50,52]. At present, two commercially available targeted NGS panels, Memorial Sloan Kettering-Integrated Mutation Profiling of Actionable Cancer Targets (MSK-IMPACT) and Foundation One CDx, each with their own panel sizes, including mutation types and bioinformatics pipelines, are FDA-approved for TMB enumeration. This variability amongst commercial panels makes comparison arduous, but the general cutoff for calling a tumor TMB-high is >10 mutations/Mb according to FDA guidelines for pembrolizumab administration [52].

## 5. Immunotherapeutic Biomarkers Per Organ System

As previously emphasized, many biomarkers are used to assess for an array of DS cancers across a wide spectrum of luminal and accessory organs. Each marker is doubly associated with idiomatic ancillary studies to be ordered and/or interpreted by pathologists to facilitate predictive and prognostic evaluations for immunotherapy that are personalized to each patient. To streamline the decision of which biomarkers should be assessed by pathologists, we compiled the pertinent biomarkers, their respective ancillary tests, and practical applications per each DS organ in Table 6, Table 7, Table 8, Table 9, Table 10 and Table 11.

Immunotherapeutic management of digestive system tumors is a burgeoning and increasingly fruitful domain of oncologic research. Major immunotherapeutic strategies that are currently used in clinical practice or the subject of promising clinical trials include ICIs, ACT, vaccines, IDOIs, and CCL2–CCR2 pathway inhibitors. In assessing whether at least one of these regimens is apropos for the patient’s cancer, pathologists are often required to interpret a plethora of predictive and prognostic biomarkers, of which the most commonly used in clinical practice are HER2, PD-L1, MSI, and TMB through a combination of ancillary tests including IHC, ISH, PCR, and NGS. We reviewed and compiled a streamlined list of clinically actionable biomarkers per major tumors from each digestive system organ with the aim of facilitating clinicians’ and pathologists’ decisions of which markers to employ and how to interpret each to guide personalized treatment for their patients.

## 6. Conclusions

Immunotherapy has introduced new and exciting perspectives regarding the management of DS cancers. However, further investigation is required to close the gap between the goal of precision therapy versus clinical practice. Our manuscript is an attempt to systematically review the progress made toward that goal and to provide a thorough understanding of the biomarkers pertinent to the prediction of patient responses. The identification and development of novel predictive immunotherapeutic biomarkers will expand treatment options for patients with advanced disease who might suffer a poor quality of life or receive no improvement in their condition under other therapeutic strategies.

## Figures and Tables

**Table 1:** Summary of HER2 immunohistochemistry (IHC) interpretation criteria in gastroesophageal adenocarcinoma.

SCORE	STAINING PATTERN (S)	INTERPRETATION
0	No reactivity OR Membranous staining in <10% of tumor cells	Negative—no ISH required
1+	Faint staining in >10% of tumor cells Reactivity limited to only part of membrane	Negative—no ISH required
2+	Weak to moderate complete, basolateral or lateral membranous staining in ≥10% of tumor cells	Equivocal—order ISH testing
3+	Strong complete, basolateral or lateral membranous staining in ≥10% of tumor cells	Positive—no ISH required

**Table 2:** Summary of HER2 immunohistochemistry (IHC) interpretation criteria in esophageal squamous cell carcinoma (ESCC).

SCORE	STAINING PATTERN (S)	INTERPRETATION
0	No reactivity OR Weak/partial staining in <10% of tumor cells	Negative—no ISH required
1+	Weak membranous staining in >10% of tumor cells	Negative—no ISH required
2+	Weak to moderate complete membranous staining in >10% of tumor cells	Equivocal—order ISH testing
3+	Strong complete membranous staining in >10% of tumor cells	Positive—no ISH required

**Table 3:** Summary of HER2 in situ hybridization (ISH) results in gastroesophageal cancers.

HER2/CEP17 RATIO	HER2 SIGNALS/CELL	INTERPRETATION
<2.0	<4.0	Negative
<2.0	≥4.0 to <6.0	Equivocal
<2.0	≥6.0	Positive
≥2.0	≥4.0 to <6.0	
≥2.0	<4.0	

**Table 4:** Recommendations for PD-L1 TPS and CPS interpretation in gastroesophageal cancers.

SUBTYPE	CPS/TPS FOR PEMBROLIZUMAB	CPS/TPS FOR NIVOLUMAB
Esophageal/GEJ adenocarcinoma	CPS ≥ 10	CPS ≥ 5
Esophageal SCC	CPS ≥ 10	TPS ≥ 1%
Gastric adenocarcinoma	No recommendations	CPS ≥ 5

**Table 5:** Common pitfalls of mismatch repair (MMR) testing per IHC.

Preanalytical factors (e.g., type of fixative used and amount of time in fixative)
Diffuse, focal staining
Lack of internal positive control
Cytoplasm-restricted staining
Missense mutation with retained protein antigenicity (nuclear staining)
MLH1 (obligatory partner) binds another secondary protein such as MSH3
MSH6 staining lost following neoadjuvant chemotherapy

**Table 6:** Immunotherapeutic biomarkers of esophageal and gastroesophageal junction (GEJ) cancers.

BIOMARKER	ANCILLARY TEST (S)	APPLICATION (S)
HER2	IHC, FISH	HER2^+^ esophageal cancers may be managed with HER2 inhibitors such as trastuzumab or pembrolizumab [40]
PD-L1	IHC	Adenocarcinomas with CPS ≥5 and ≥10 approved for treatment with nivolumab and pembrolizumab, respectively [40] ESCC with TPS ≥ 1% and CPS ≥ 10 approved for treatment with nivolumab and pembrolizumab, respectively [40]
MSI	IHC, PCR, NGS	Pembrolizumab approved for management of MSI-H solid tumors [55]
TMB	NGS	Pembrolizumab approved for all solid tumors with TMB ≥ 10 Mut/Mb [52]

**Table 7:** Immunotherapeutic biomarkers of gastric cancers.

BIOMARKER	ANCILLARY TEST (S)	APPLICATION (S)
HER2	IHC, FISH	HER2^+^ gastric cancer may be treated with HER2 inhibitors such as trastuzumab
PD-L1	IHC	CPS ≥ 5: Approved for treatment with HER2 inhibitor trastuzumab [44,56,57]
MSI	IHC, PCR, NGS	Pembrolizumab approved for management of MSI-H solid tumors [55]
TMB	NGS	Pembrolizumab approved for all solid tumors with TMB ≥ 10 Mut/Mb [52]
EBV	IHC, PCR	EBV expression associated with increased tumor-infiltrating lymphocytes (TILs) and increased response to ICIs [56,57]
LAG3	IHC	LAG3 expression may indicate treatment with novel LAG3 inhibitor relatlimab [58]
MUC1	IHC	Positive (cytoplasmic) staining may indicate CAR-T therapy [59,60]
CEA	IHC	Possible CAR-T target [59]
EPCAM	IHC	EpCAM inhibitor catumaxomab may be used to treat peritoneal carcinomatosis of EpCAm^+^ gastric cancer [61,62]
MESOTHELIN	IHC	Positive staining for mesothelin may indicate CAR-T therapy [59]
CLDN 18.2	IHC	Positive staining for CLDN 18.2 may indicate treatment with CL18.2 inhibitor zolbetuximab or CAR-T therapy [59,63]

Abbreviations: Epstein–Barr virus (EBV); lymphocyte-activation gene 3 (LAG3); carcinoembryonic antigen (CEA); epithelial cell adhesion molecule (EpCAM); Claudin 18.2 (CLDN 18.2).

**Table 8:** Immunotherapeutic biomarkers of intestinal cancers.

BIOMARKER	ANCILLARY TEST (S)	APPLICATION (S)
MSI	IHC, PCR, NGS	Pembrolizumab approved for management of MSI-H CRC [55]
TMB	NGS	Pembrolizumab approved for all solid tumors with TMB ≥ 10 Mut/Mb [52]
CEA	IHC	CEA-T cell specific antibody (CEA-TCB) [64]
B2M	PCR	MSI-H patients with mutations of the gene encoding this protein may be resistant to management by ICIs [65]
B-CATENIN	IHC	Nuclear β-catenin inversely proportional to TIL density → poor response to ICIs → may be overcome by ICRT14 β-catenin inhibitor [66]
*PTEN*	IHC, PCR, NGS	Loss of PTEN expression associated with reduced CD8^+^ TILs → poor response to ICIs [67]
*JAK1/2*	PCR, NGS	JAK1/2 mutations associated with ICI resistance in TMB-high CRC [68]
*BRAF*	PCR, NGS	BRAF V600E mutations associated with poor response to chemotherapy and increased response to ICIs [69] BRAF K601E mutations associated with better response to chemotherapy [69]
*KRAS*	PCR, NGS	KRAS mutations indicative of primary resistance to immunotherapy [70]
CD8+ TILS	IHC	High CD8^+^ TIL count associated with good response to ICIs [65]
CD39+ TILS	IHC	High CD39^+^ TIL count associated with good response to ICIs [71]
*POLE/POLD1*	PCR, NGS	POLE and POLD1 mutations associated with MSI, high TMB, high PD-L1 expression, and increased TILs → Good response to ICIs [72]

Abbreviations: beta-2 microglobulin (B2M).

**Table 9:** Immunotherapeutic biomarkers of pancreatic cancers.

BIOMARKER	ANCILLARY TEST (S)	APPLICATION (S)
MSI	IHC, PCR, NGS	Pembrolizumab approved for management of MSI-H solid tumors [55]
TMB	NGS	Pembrolizumab approved for all solid tumors with TMB ≥ 10 Mut/Mb [52]
MUC1	IHC	Potential target of adoptive T-cell and dendritic cell therapy [73]
MESOTHELIN	IHC	Potential target of adoptive T-cell therapy and peptide vaccines [74]
FAP	IHC	Potential target of CAR-T therapy [75]
*BRCA1/2*	PCR, NGS	BRCA1/2-mutant pancreatic adenocarcinoma may be targeted by poly ADP ribose polymerase (PARP) inhibitor olaparib [76]
*PALB2*	PCR, NGS	PALB2-mutant pancreatic adenocarcinoma is a plausible target of olaparib [77]
*NTRK*	PCR, NGS	NTRK-mutant pancreatic adenocarcinoma may be targeted by NTRK inhibitors such as larotrectinib [78]
*KRAS*	PCR, NGS	KRAS-mutant pancreatic adenocarcinoma may be associated with poor response to ICIs [79]
*NRG1*	PCR, NGS	NRG1-mutant pancreatic adenocarcinoma may be targeted by NRG1 inhibitor zenocutuzumab [80]

Abbreviations: fibroblast activation protein (FAP).

**Table 10:** Immunotherapeutic biomarkers of hepatobiliary cancers.

	BIOMARKER	ANCILLARY TEST (S)	APPLICATION (S)
HEPATOCELULAR CARCINOMA (HCC)	MSI	IHC, PCR, NGS	Pembrolizumab approved for management of MSI-H solid tumors [55]
	TMB	NGS	Pembrolizumab approved for all solid tumors with TMB ≥ 10 Mut/Mb [52]
	GLY-3	IHC	Gly-3 may be targeted by CAR T-cell therapy or peptide vaccine [81,82]
	TIM3	IHC	TIM3 indicates poor response to ICIs [83,84]
	LAG3	IHC	LAG3 expression may serve as a target for LAG3 inhibitors [84]
	Beta-Catenin	IHC, PCR, NGS	Beta-catenin^+^ HCC associated with poor response to immunotherapy [85]
	TP53	IHC, PCR, NGS	Associated with poor response to immunotherapy [85]
	NKG2DL	IHC	Potential target of CAR-T therapy [86]
	AFP	IHC	Target of novel peptide vaccine [87]
	hTERT	IHC	Target of novel peptide vaccine [81]
	MRP3	IHC	Target of novel peptide vaccine [81]
	HCV	IHC	Potential target of modified TCR-T cell therapy for a subset of HCCs [88]
INTRAHEPATIC CHOLANGIO-CARCINOMA (ICAC)	MSI	IHC, PCR, NGS	Pembrolizumab approved for management of MSI-H solid tumors [55]
	TMB	NGS	Pembrolizumab approved for all solid tumors with TMB ≥ 10 Mut/Mb [52]
	IDH1/2	PCR, NGS	IDH inhibitors such as ivosidenib may be used to treat IDH-mutant ICAC [89]
	FGFR2	PCR, NGS	FGFR inhibitor pemigatinib may be used to treat FGFR-mutant ICAC [90]
	WT1	IHC	Target of dendritic cell vaccine [91]
	MUC1	IHC	Target of dendritic cell vaccine [91]
EXTRAHEPATIC CHOLANGIO-CARCINOMA (ECAC)	MSI	IHC, PCR, NGS	Pembrolizumab approved for management of MSI-H solid tumors [55]
	TMB	NGS	Pembrolizumab approved for all solid tumors with TMB ≥ 10 Mut/Mb [52]
	HER2	IHC, FISH	HER2^+^ ECAC is a potential target of HER2 inhibitors such as trastuzumab [92]
	MEK	PCR, NGS	MEK-mutant ECAC may be managed with MEK1/2 Inhibitor binimetinib [93]
GALLBLADDER CARCINOMA	MSI	IHC, PCR, NGS	Pembrolizumab approved for management of MSI-H solid tumors [55]
	TMB	NGS	Pembrolizumab approved for all solid tumors with TMB ≥ 10 Mut/Mb [52]
	HER2	IHC, FISH	HER2^+^ ECAC is a potential target of HER2 inhibitors such as trastuzumab [94]
	BRCA1/2	PCR	Strong correlation with MSI-H status and good response to ICIs [95]
	TP53	IHC, PCR	Confers poor prognosis and may be target of intralesional ONYX-015 oncolytic adenovirus [96]

Abbreviations: Glypican-3 (GLY-3); T-cell immunoglobulin and mucin domain-containing protein 3 (TIM3); natural killer group 2, member D ligand (NKG2DL); alpha-fetoprotein (AFP); human telomerase reverse transcriptase (hTERT); multidrug resistance protein 3 (MRP3); hepatitis C virus (HCV); Wilms tumor 1 (WT1).

**Table 11:** Immunotherapeutic biomarkers of other miscellaneous cancers.

	BIOMARKER	ANCILLARY TEST (S)	APPLICATION (S)
ANAL SQUAMOUS CELL CARCINOMA	MSI	IHC, PCR, NGS	Pembrolizumab approved for management of MSI-H solid tumors [55]
	TMB	NGS	Pembrolizumab approved for all solid tumors with TMB ≥ 10 Mut/Mb [52]
	P16	IHC	Epitopes of HPV16 E6 and E7 proteins are potential targets of novel vaccines and adoptive T-cell therapy [97]
GASTROINTESTINAL STROMAL TUMOR (GIST)	MSI	IHC, PCR, NGS	Pembrolizumab approved for management of MSI-H solid tumors [55]
	TMB	NGS	Pembrolizumab approved for all solid tumors with TMB ≥ 10 Mut/Mb [52]
	*PDGFRA*	PCR, NGS	PDGFRA D842V mutation confers increased response to ICIs [98]
	*cKIT/CD117*	IHC, PCR, NGS	cKIT inhibitor imatinib exhibits immunotherapeutic effects via inhibition of IDO [99]
NEUROENDOCRINE TUMOR (NET)	MSI	IHC, PCR, NGS	Pembrolizumab approved for management of MSI-H solid tumors [55]
	TMB	NGS	Pembrolizumab approved for all solid tumors with TMB ≥ 10 Mut/Mb [52]
	CDH17	IHC	Possible target of CAR-T therapy [100]
	SSTR2/5	IHC	Possible target of CAR-T Therapy [100]

Abbreviations: platelet-derived growth factor receptor alpha (PDGFRA); Cadherin 17 (CDH17); somatostatin receptor 2/5 (SSTR2/5).

## References

[1] Arnold M., Abnet C.C., Neale R.E., Vignat J., Giovannucci E.L., McGlynn K.A., Bray F. (2020). Global Burden of 5 Major Types of Gastrointestinal Cancer. Gastroenterology.

[2] Siegel R.L., Miller K.D., Fuchs H.E., Jemal A. (2022). Cancer statistics, 2022. CA Cancer J. Clin..

[3] Yang S., Liu T., Cheng Y., Bai Y., Liang G. (2019). Immune cell infiltration as a biomarker for the diagnosis and prognosis of digestive system cancer. Cancer Sci..

[4] Nagtegaal I.D., Odze R.D., Klimstra D., Paradis V., Rugge M., Schirmacher P., Washington K.M., Carneiro F., Cree I.A., WHO Classification of Tumours Editorial Board (2020). The 2019 WHO classification of tumours of the digestive system. Histopathology.

[5] Marin-Acevedo J.A., Kimbrough E.M.O., Lou Y. (2021). Next generation of immune checkpoint inhibitors and beyond. J. Hematol. Oncol..

[6] Johdi N.A., Sukor N.F. (2020). Colorectal Cancer Immunotherapy: Options and Strategies. Front. Immunol..

[7] Shiravand Y., Khodadadi F., Kashani S.M.A., Hosseini-Fard S.R., Hosseini S., Sadeghirad H., Ladwa R., O’Byrne K., Kulasinghe A. (2022). Immune Checkpoint Inhibitors in Cancer Therapy. Curr. Oncol..

[8] Rohaan M.W., Wilgenhof S., Haanen J.B.A.G. (2019). Adoptive cellular therapies: The current landscape. Virchows Arch..

[9] Li J., Li W., Huang K., Zhang Y., Kupfer G., Zhao Q. (2018). Chimeric antigen receptor T cell (CAR-T) immunotherapy for solid tumors: Lessons learned and strategies for moving forward. J. Hematol. Oncol..

[10] Magee M.S., Abraham T.S., Baybutt T.R., Flickinger J.C., Ridge N.A., Marszalowicz G.P., Prajapati P., Hersperger A.R., Waldman S.A., Snook A.E. (2018). Human GUCY2C-targeted chimeric antigen receptor (CAR)-expressing T cells eliminate colorectal cancer metastases. Cancer Immunol. Res..

[11] Siegler E.L., Zhu Y., Wang P., Yang L. (2018). Off-the-Shelf CAR-NK Cells for Cancer Immunotherapy. Cell Stem Cell.

[12] Jäkel C.E., Vogt A., Gonzalez-Carmona M.A., Schmidt-Wolf I.G.H. (2014). Clinical studies applying cytokine-induced killer cells for the treatment of gastrointestinal tumors. J. Immunol. Res..

[13] Rahma O.E., Khleif S.N. (2011). Therapeutic vaccines for gastrointestinal cancers. Gastroenterol. Hepatol..

[14] Chudasama R., Phung Q., Hsu A., Almhanna K. (2021). Vaccines in gastrointestinal malignancies: From prevention to treatment. Vaccines.

[15] Liu J., Fu M., Wang M., Wan D., Wei Y., Wei X. (2022). Cancer vaccines as promising immuno-therapeutics: Platforms and current progress. J. Hematol. Oncol..

[16] Pan L., Shang N., Shangguan J., Figini M., Xing W., Wang B., Sun C., Yang J., Zhang Y., Hu S. (2019). Magnetic resonance imaging monitoring therapeutic response to dendritic cell vaccine in murine orthotopic pancreatic cancer models. Am. J. Cancer Res..

[17] Fujiwara Y., Kato S., Nesline M.K., Conroy J.M., DePietro P., Pabla S., Kurzrock R. (2022). Indoleamine 2,3-dioxygenase (IDO) inhibitors and cancer immunotherapy. Cancer Treat. Rev..

[18] Prendergast G.C., Malachowski W.J., Mondal A., Scherle P., Muller A.J. (2018). Indoleamine 2,3-Dioxygenase and Its Therapeutic Inhibition in Cancer. Int. Rev. Cell Mol. Biol..

[19] Zhang R., Li T., Wang W., Gan W., Lv S., Zeng Z., Hou Y., Yan Z., Yang M. (2020). Indoleamine 2, 3-Dioxygenase 1 and CD8 Expression Profiling Revealed an Immunological Subtype of Colon Cancer with a Poor Prognosis. Front. Oncol..

[20] Fei L., Ren X., Yu H., Zhan Y. (2021). Targeting the CCL2/CCR2 Axis in Cancer Immunotherapy: One Stone, Three Birds?. Front. Immunol..

[21] Dagouassat M., Suffee N., Hlawaty H., Haddad O., Charni F., Laguillier C., Vassy R., Martin L., Schischmanoff P., Gattegno L. (2010). Monocyte chemoattractant protein-1 (MCP-1)/CCL2 secreted by hepatic myofibroblasts promotes migration and invasion of human hepatoma cells. Int. J. Cancer.

[22] Monti P., Leone B.E., Marchesi F., Balzano G., Zerbi A., Scaltrini F., Pasquali C., Calori G., Pessi F., Sperti C. (2003). The CC chemokine MCP-1/CCL2 in pancreatic cancer progression: Regulation of expression and potential mechanisms of antimalignant activity. Cancer Res..

[23] Xu W., Wei Q., Han M., Zhou B., Wang H., Zhang J., Wang Q., Sun J., Feng L., Wang S. (2018). CCL2-SQSTM1 positive feedback loop suppresses autophagy to promote chemoresistance in gastric cancer. Int. J. Biol. Sci..

[24] Ou B., Cheng X., Xu Z., Chen C., Shen X., Zhao J., Lu A. (2019). A positive feedback loop of β-catenin/CCR2 axis promotes regorafenib resistance in colorectal cancer. Cell Death Dis..

[25] Nywening T.M., Wang-Gillam A., Sanford D.E., Belt B.A., Panni R.Z., Cusworth B.M., Toriola A.T., Nieman R.K., Worley L.A., Yano M. (2016). Targeting tumour-associated macrophages with CCR2 inhibition in combination with FOLFIRINOX in patients with borderline resectable and locally advanced pancreatic cancer: A single-centre, open-label, dose-finding, non-randomised, phase 1b trial. Lancet Oncol..

[26] Connolly K.A., Belt B.A., Figueroa N.M., Murthy A., Patel A., Kim M., Lord E.M., Linehan D.C., Gerber S. (2016). Increasing the efficacy of radiotherapy by modulating the CCR2/CCR5 chemokine axes. Oncotarget.

[27] Duraiyan J., Govindarajan R., Kaliyappan K., Palanisamy M. (2012). Applications of immunohistochemistry. J. Pharm. Bioallied Sci..

[28] Rizk E.M., Gartrell R.D., Barker L.W., Esancy C.L., Finkel G.G., Bordbar D.D., Saenger Y.M. (2019). Prognostic and Predictive Immunohistochemistry-Based Biomarkers in Cancer and Immunotherapy. Hematol. Oncol. Clin. N. Am..

[29] Cui C., Shu W., Li P. (2016). Fluorescence In Situ Hybridization: Cell-Based Genetic Diagnostic and Research Applications. Front. Cell Dev. Biol..

[30] Kwon S. (2013). Single-molecule fluorescence in situ hybridization: Quantitative imaging of single RNA molecules. BMB Rep..

[31] Matsuda K. (2017). PCR-Based Detection Methods for Single-Nucleotide Polymorphism or Mutation: Real-Time PCR and Its Substantial Contribution Toward Technological Refinement. Adv. Clin. Chem..

[32] Behjati S., Tarpey P.S. (2013). What is next generation sequencing?. Arch. Dis. Child.-Educ. Pract. Ed..

[33] Nagahashi M., Shimada Y., Ichikawa H., Kameyama H., Takabe K., Okuda S., Wakai T. (2019). Next generation sequencing-based gene panel tests for the management of solid tumors. Cancer Sci..

[34] Prokop J.W., May T., Strong K., Bilinovich S.M., Bupp C., Rajasekaran S., Worthey E.A., Lazar J. (2018). Genome sequencing in the clinic: The past, present, and future of genomic medicine. Physiol. Genom..

[35] Lincoln S.E., Nussbaum R.L., Kurian A.W., Nielsen S.M., Das K., Michalski S., Yang S., Ngo N., Blanco A., Esplin E.D. (2020). Yield and Utility of Germline Testing Following Tumor Sequencing in Patients With Cancer. JAMA Netw. Open.

[36] Bewicke-Copley F., Arjun Kumar E., Palladino G., Korfi K., Wang J. (2019). Applications and analysis of targeted genomic sequencing in cancer studies. Comput. Struct. Biotechnol. J..

[37] Bartley A.N., Washington M.K., Colasacco C., Ventura C.B., Ismaila N., Benson III A.B., Carrato A., Gulley M.L., Jain D., Kakar S. (2017). HER2 Testing and Clinical Decision Making in Gastroesophageal Adenocarcinoma: Guideline From the College of American Pathologists, American Society for Clinical Pathology, and the American Society of Clinical Oncology. J. Clin. Oncol..

[38] Rong L., Wang B., Guo L., Liu X., Wang B., Ying J., Xue L., Lu N. (2020). HER2 expression and relevant clinicopathological features in esophageal squamous cell carcinoma in a Chinese population. Diagn. Pathol..

[39] Creemers A., Ebbing E.A., Hooijer G.K.J., Stap L., Jibodh-Mulder R.A., Gisbertz S.S., van Berge Henegouwen M.I., van Montfoort M.L., Hulshof M.C.C.M., Krishnadath K.K. (2018). The dynamics of HER2 status in esophageal adenocarcinoma. Oncotarget.

[40] Patruni S., Fayyaz F., Bien J., Phillip T., King D.A. (2022). Immunotherapy in the Management of Esophagogastric Cancer: A Practical Review. JCO Oncol. Pract.

[41] Tabernero J., Bang Y.J., van Cutsem E., Fuchs C.S., Janjigian Y.Y., Bhagia P., Li K., Adelberg D., Qin S.K. (2021). KEYNOTE-859: A Phase III study of pembrolizumab plus chemotherapy in gastric/gastroesophageal junction adenocarcinoma. Future Oncol..

[42] Mastracci L., Grillo F., Parente P., Gullo I., Campora M., Angerilli V., Rossi C., Sacramento M.L., Pennelli G., Vanoli A. (2022). PD-L1 evaluation in the gastrointestinal tract: From biological rationale to its clinical application. Pathologica.

[43] Akhtar M., Rashid S., Al-Bozom I.A. (2021). PD-L1 immunostaining: What pathologists need to know. Diagn. Pathol..

[44] Luchini C., Bibeau F., Ligtenberg M.J.L., Singh N., Nottegar A., Bosse T., Miller R., Riaz N., Douillard J.-Y., Andre F. (2019). ESMO recommendations on microsatellite instability testing for immunotherapy in cancer, and its relationship with PD-1/PD-L1 expression and tumour mutational burden: A systematic review-based approach. Ann. Oncol..

[45] Sun B.L. (2021). Current Microsatellite Instability Testing in Management of Colorectal Cancer. Clin. Color. Cancer.

[46] Quaas A., Rehkaemper J., Rueschoff J., Pamuk A., Zander T., Hillmer A., Siemanowski J., Wittig J., Buettner R., Plum P. (2021). Occurrence of High Microsatellite-Instability/Mismatch Repair Deficiency in Nearly 2000 Human Adenocarcinomas of the Gastrointestinal Tract, Pancreas, and Bile Ducts: A Study From a Large German Comprehensive Cancer Center. Front. Oncol..

[47] Shia J. (2008). Immunohistochemistry versus microsatellite instability testing for screening colorectal cancer patients at risk for hereditary nonpolyposis colorectal cancer syndrome: Part I. The utility of immunohistochemistry. J. Mol. Diagn..

[48] Benhamida J.K., Hechtman J.F., Nafa K., Villafania L., Sadowska J., Wang J., Wong D., Zehir A., Zhang L., Bale T. (2020). Reliable Clinical MLH1 Promoter Hypermethylation Assessment Using a High-Throughput Genome-Wide Methylation Array Platform. J. Mol. Diagn..

[49] Chen W., Frankel W.L. (2019). A practical guide to biomarkers for the evaluation of colorectal cancer. Mod. Pathol..

[50] Engel K.B., Moore H.M. (2011). Effects of Preanalytical Variables on the Detection of Proteins by Immunohistochemistry in Formalin-Fixed, Paraffin-Embedded Tissue. Arch. Pathol. Lab. Med..

[51] Vanderwalde A., Spetzler D., Xiao N., Gatalica Z., Marshall J. (2018). Microsatellite instability status determined by next-generation sequencing and compared with PD-L1 and tumor mutational burden in 11,348 patients. Cancer Med..

[52] Sha D., Jin Z., Budczies J., Kluck K., Stenzinger A., Sinicrope F.A. (2020). Tumor mutational burden as a predictive biomarker in solid tumors. Cancer Discov..

[53] Goodman Z.D. (2017). Phenotypes and Pathology of Drug-Induced Liver Disease. Clin. Liver Dis..

[54] Salem M.E., Bodor J.N., Puccini A., Xiu J., Goldberg R.M., Grothey A., Korn W.M., Shields A.F., Worrilow W.M., Kim E.S. (2020). Relationship between MLH1, PMS2, MSH2 and MSH6 gene-specific alterations and tumor mutational burden in 1057 microsatellite instability-high solid tumors. Int. J. Cancer.

[55] Lemery S., Keegan P., Pazdur R. (2017). First FDA Approval Agnostic of Cancer Site—When a Biomarker Defines the Indication. N. Engl. J. Med..

[56] Chang X., Ge X., Zhang Y., Xue X. (2022). The current management and biomarkers of immunotherapy in advanced gastric cancer. Medicine.

[57] Takei S., Kawazoe A., Shitara K. (2022). The New Era of Immunotherapy in Gastric Cancer. Cancers.

[58] Ohmura H., Yamaguchi K., Hanamura F., Ito M., Makiyama A., Uchino K., Shimokawa H., Tamura S., Esaki T., Mitsugi K. (2020). OX40 and LAG3 are associated with better prognosis in advanced gastric cancer patients treated with anti-programmed death-1 antibody. Br. J. Cancer.

[59] Bębnowska D., Grywalska E., Niedźwiedzka-Rystwe P., Sosnowska-Pasiarska B., Smok-Kalwat J., Pasiarski M., Gozdz S., Rolinski J., Polkowski W. (2020). CAR-T cell therapy—An Overview of Targets in Gastric Cancer. J. Clin. Med..

[60] Hwang I., Kang Y.N., Kim J.Y., Do Y.R., Song H.S., Park K.U. (2012). Prognostic significance of membrane-associated mucins 1 and 4 in gastric adenocarcinoma. Exp. Ther. Med..

[61] Knödler M., Körfer J., Kunzmann V., Trojan J., Daum S., Schenk M., Kullmann F., Schroll S., Behringer D., Stahl M. (2018). Randomised phase II trial to investigate catumaxomab (anti-EpCAM × anti-CD3) for treatment of peritoneal carcinomatosis in patients with gastric cancer. Br. J. Cancer.

[62] Faghfuri E., Shadbad M.A., Faghfouri A.H., Soozangar N. (2022). Cellular immunotherapy in gastric cancer: Adoptive cell therapy and dendritic cell-based vaccination. Immunotherapy.

[63] Sahin U., Türeci O., Manikhas G., Lordick F., Rusyn A., Vynnychenko I., Dudov A., Bazin I., Bondarenko I., Melichar B. (2021). FAST: A randomised phase II study of zolbetuximab (IMAB362) plus EOX versus EOX alone for first-line treatment of advanced CLDN18.2-positive gastric and gastro-oesophageal adenocarcinoma. Ann. Oncol..

[64] Tabernero J., Melero I., Ros W., Argiles G., Marabelle A., Rodriguez-Ruiz M.E., Albanell J., Calvo E., Moreno V., Cleary J.M. (2017). Phase Ia and Ib studies of the novel carcinoembryonic antigen (CEA) T-cell bispecific (CEA CD3 TCB) antibody as a single agent and in combination with atezolizumab: Preliminary efficacy and safety in patients with metastatic colorectal cancer (mCRC). J. Clin. Oncol..

[65] Weng J., Li S., Zhu Z., Liu Q., Zhang R., Yang Y., Li X. (2022). Exploring immunotherapy in colorectal cancer. J. Hematol. Oncol..

[66] Wang C., Yan J., Yin P., Gui L., Ji L., Ma B., Gao W. (2020). β-Catenin inhibition shapes tumor immunity and synergizes with immunotherapy in colorectal cancer. Oncoimmunology.

[67] Hu-Lieskovan S., Mok S., Moreno B.H., Tsoi J., Robert L., Goedert L., Pinheiro E.M., Koya R.C., Graeber T.G., Comin-Anduix B. (2015). Improved antitumor activity of immunotherapy with BRAF and MEK inhibitors in BRAF(V600E) melanoma. Sci. Transl. Med..

[68] Patel S.J., Sanjana N.E., Kishton R.J., Eidizadeh A., Vodnala S.K., Cam M., Gartner J.J., Jia L., Steinberg S.M., Yamamoto T.N. (2017). Identification of essential genes for cancer immunotherapy. Nature.

[69] Ciombor K.K., Strickler J.H., Bekaii-Saab T.S., Yaeger R. (2022). *BRAF*-Mutated Advanced Colorectal Cancer: A Rapidly Changing Therapeutic Landscape. J. Clin. Oncol..

[70] Lal N., White B.S., Goussous G., Pickles O., Mason M.J., Beggs A.D., Taniere P., Wilcox B.E., Guinney J., Middleton G.W. (2018). KRAS mutation and consensus molecular subtypes 2 and 3 are independently associated with reduced immune infiltration and reactivity in colorectal cancer. Clin. Cancer Res..

[71] Krishna S., Lowery F.J., Copeland A.R., Bahadiroglu E., Mukherjee R., Jia L., Anibal J.T., Sachs A., Adebola S.O., Gurusamy D. (2020). Stem-like CD8 T cells mediate response of adoptive cell immunotherapy against human cancer. Science.

[72] Wang F., Zhao Q., Wang Y.N., Jin Y., He M., Liu Z., Xu R. (2019). Evaluation of POLE and POLD1 Mutations as Biomarkers for Immunotherapy Outcomes Across Multiple Cancer Types. JAMA Oncol..

[73] Shindo Y., Hazama S., Maeda Y., Matsui H., Iida M., Suzuki N., Yoshimura Y., Ueno T., Yoshino S., Sakai K. (2014). Adoptive immunotherapy with MUC1-mRNA transfected dendritic cells and cytotoxic lymphocytes plus gemcitabine for unresectable pancreatic cancer. J. Transl. Med..

[74] Le K., Wang J., Zhang T., Guo Y., Chang H., Wang S., Zhu B. (2020). Overexpression of mesothelin in pancreatic ductal adenocarcinoma (PDAC). Int. J. Med. Sci..

[75] DeSelm C.J., Tano Z.E., Varghese A.M., Adusumilli P.S. (2017). CAR T-cell therapy for pancreatic cancer. J. Surg. Oncol..

[76] Rosen M.N., Goodwin R.A., Vickers M.M. (2021). BRCA mutated pancreatic cancer: A change is coming. World J. Gastroenterol..

[77] Wu W., Liu Y., Jin Y., Liu L., Guo Y., Xu M., Hao Q., Li D., Fang W., Zhang A. (2022). Case Report: Effectiveness of Targeted Treatment in a Patient With Pancreatic Cancer Harboring PALB2 Germline Mutation and KRAS Somatic Mutation. Front. Med..

[78] O’Reilly E.M., Hechtman J.F. (2019). Tumour response to TRK inhibition in a patient with pancreatic adenocarcinoma harbouring an NTRK gene fusion. Ann. Oncol..

[79] Di Federico A., Mosca M., Pagani R., Carloni R., Frega G., De Giglio A., Rizzo A., Ricci D., Tavolari S., Di Marco M. (2022). Immunotherapy in Pancreatic Cancer: Why Do We Keep Failing? A Focus on Tumor Immune Microenvironment, Predictive Biomarkers and Treatment Outcomes. Cancers.

[80] Schram A.M., Odintsov I., Espinosa-Cotton M., Khodos I., Sisso W.J., Mattar M.S., Lui A.J.W., Vojnic M., Shameem S.H., Chauhan T. (2022). Zenocutuzumab, a HER2xHER3 Bispecific Antibody, Is Effective Therapy for Tumors Driven by NRG1 Gene Rearrangements. Cancer Discov..

[81] Liu Z., Liu X., Liang J., Liu Y., Hou X., Zhang M., Li Y., Jiang X. (2021). Immunotherapy for Hepatocellular Carcinoma: Current Status and Future Prospects. Front. Immunol..

[82] Shinoda M., Kishida N., Itano O., Shigenori E., Ueno A., Kitago M., Abe Y., Hibi T., Yagi H., Masugi Y. (2015). Long-term complete response of advanced hepatocellular carcinoma treated with multidisciplinary therapy including reduced dose of sorafenib: Case report and review of the literature. World J. Surg. Oncol..

[83] Li H., Wu K., Tao K., Chen L., Zheng Q., Lu X., Liu J., Shi L., Liu C., Wang G. (2012). Tim-3/galectin-9 signaling pathway mediates T-cell dysfunction and predicts poor prognosis in patients with hepatitis B virus-associated hepatocellular carcinoma. Hepatology.

[84] Guo M., Yuan F., Qi F., Sun J., Rao Q., Zhao Z., Huang P., Fang T., Yang B., Xia J. (2020). Expression and clinical significance of LAG-3, FGL1, PD-L1 and CD8^+^T cells in hepatocellular carcinoma using multiplex quantitative analysis. J. Transl. Med..

[85] He Y., Lu M., Che J., Chu Q., Zhang P., Chen Y. (2021). Biomarkers and Future Perspectives for Hepatocellular Carcinoma Immunotherapy. Front. Oncol..

[86] Sun B., Yang D., Dai H., Liu X., Jia R., Cui X., Li W., Cai C., Xu J., Zhao X. (2019). Eradication of hepatocellular carcinoma by NKG2D-based CAR-T cells. Cancer Immunol. Res..

[87] Nakagawa H., Mizukoshi E., Kobayashi E., Tamai T., Hamana H., Ozawa T., Kishi H., Kitahara M., Yamashita T., Arai K. (2017). Association Between High-Avidity T-Cell Receptors, Induced by α-Fetoprotein−Derived Peptides, and Anti-Tumor Effects in Patients With Hepatocellular Carcinoma. Gastroenterology.

[88] Spear T.T., Callender G.G., Roszkowski J.J., Moxley K.M., Simms P.E., Foley K.C., Murray D.C., Scurti G.M., Li M., Thomas J.T. (2016). TCR gene-modified T cells can efficiently treat established hepatitis C-associated hepatocellular carcinoma tumors. Cancer Immunol. Immunother..

[89] Lamarca A., Barriuso J., McNamara M.G., Valle J.W. (2020). Molecular targeted therapies: Ready for “prime time” in biliary tract cancer. J. Hepatol..

[90] Li Y., Song Y., Liu S. (2022). The New Insight of Treatment in Cholangiocarcinoma. J. Cancer.

[91] Kobayashi M., Sakabe T., Abe H., Tanii M., Takahashi H., Chiba A., Yanagida E., Shibamoto Y., Ogasawara M., Tsujitani S. (2013). Dendritic Cell-Based Immunotherapy Targeting Synthesized Peptides for Advanced Biliary Tract Cancer. J. Gastrointest. Surg..

[92] Hong Y., Li X., Cao D. (2021). Case Report: Trastuzumab Treatment in Adenosquamous Carcinoma of the Extrahepatic Biliary Tract With Her-2 Amplification. Front. Oncol..

[93] Kim J.W., Lee K., Kim J.W., Sun K.J., Nam A., Bang J., Bang Y., Oh D. (2019). Enhanced Antitumor Effect of Binimetinib in Combination With Capecitabine for Biliary Tract Cancer Patients With Mutations in the RAS/RAF/MEK/ERK Pathway: Phase Ib Study. Br. J. Cancer.

[94] Javle M., Borad M.J., Azad N.S., Kurzrock R., Abou-Alfa G.K., George B., Hainsworth J., Meric-Bernstam F., Swanton C., Sweeney C.J. (2021). Pertuzumab and Trastuzumab for HER2-Positive, Metastatic Biliary Tract Cancer (MyPathway): A multicentre, open-label, phase 2a, multiple basket study. Lancet Oncol..

[95] Song X., Hu Y., Li Y., Shao R., Liu F., Liu Y. (2020). Overview of current targeted therapy in gallbladder cancer. Signal Transduct. Target. Ther..

[96] Makower D., Rozenblit A., Kaufman H., Edelman M., Lane M.E., Zwiebel J., Haynes H., Wadler S. (2003). Phase II clinical trial of intralesional administration of the oncolytic adenovirus ONYX-015 in patients with hepatobiliary tumors with correlative p53 studies. Clin. Cancer Res..

[97] Saúde-Conde R., Parisi A., Giunta E.F., Meyers M., Sclafani F. (2022). The emerging role of immunotherapy in the treatment of anal cancer. Curr. Opin. Pharmacol..

[98] Indio V., Astolfi A., Urbini M., Nannini M., Pantaleo M.A. (2020). Genetics and treatment of gastrointestinal stromal tumors with immune checkpoint inhibitors: What do we know?. Pharmacogenomics.

[99] Arshad J., Costa P.A., Barreto-Coelho P., Valdes B.N., Trent J.C. (2021). Immunotherapy Strategies for Gastrointestinal Stromal Tumor. Cancers.

[100] Fanciulli G., Modica R., La Salvia A., Campolo F., Florio T., Mikovic N., Plebani A., Di Vito V., Colao A., Faggiano A. (2022). Immunotherapy of Neuroendocrine Neoplasms: Any Role for the Chimeric Antigen Receptor T Cells?. Cancers.

